# MAHMI database: a comprehensive MetaHit-based resource for the study of the mechanism of action of the human microbiota

**DOI:** 10.1093/database/baw157

**Published:** 2017-01-10

**Authors:** Aitor Blanco-Míguez, Alberto Gutiérrez-Jácome, Florentino Fdez-Riverola, Anália Lourenço, Borja Sánchez

**Affiliations:** 1 ESEI – Department of Computer Science, University of Vigo, Edificio Politécnico, Campus Universitario As Lagoas s/n 32004, Ourense, Spain; 2 Department of Microbiology and Biochemistry of Dairy Products, Instituto de Productos Lácteos de Asturias (IPLA), Consejo Superior de Investigaciones Científicas (CSIC), Villaviciosa, Asturias, Spain; 3 CEB – Centre of Biological Engineering, University of Minho, Campus de Gualtar, 4710-057 Braga, Portugal

## Abstract

The Mechanism of Action of the Human Microbiome (MAHMI) database is a unique resource that provides comprehensive information about the sequence of potential immunomodulatory and antiproliferative peptides encrypted in the proteins produced by the human gut microbiota. Currently, MAHMI database contains over 300 hundred million peptide entries, with detailed information about peptide sequence, sources and potential bioactivity. The reference peptide data section is curated manually by domain experts. The *in silico* peptide data section is populated automatically through the systematic processing of publicly available exoproteomes of the human microbiome. Bioactivity prediction is based on the global alignment of the automatically processed peptides with experimentally validated immunomodulatory and antiproliferative peptides, in the reference section. MAHMI provides researchers with a comparative tool for inspecting the potential immunomodulatory or antiproliferative bioactivity of new amino acidic sequences and identifying promising peptides to be further investigated. Moreover, researchers are welcome to submit new experimental evidence on peptide bioactivity, namely, empiric and structural data, as a proactive, expert means to keep the database updated and improve the implemented bioactivity prediction method. Bioactive peptides identified by MAHMI have a huge biotechnological potential, including the manipulation of aberrant immune responses and the design of new functional ingredients/foods based on the genetic sequences of the human microbiome. Hopefully, the resources provided by MAHMI will be useful to those researching gastrointestinal disorders of autoimmune and inflammatory nature, such as Inflammatory Bowel Diseases. MAHMI database is routinely updated and is available free of charge.

**Database URL:** http://mahmi.org/

## Introduction

Human beings live in a homeostatic symbiosis with their gastrointestinal microbes (1). The host provides microbiota with nutrients and a friendly environment, whereas the microbiota helps in the development of the gut mucosa, including the immune system, and provides the host with nutritional contributions (2). Different functions have been assigned to human gut microbiota, including maintenance of the epithelial barrier, direct or indirect inhibition of pathogen adhesion to intestinal surfaces, modulation and proper maturation of the immune system, degradation of otherwise non-digestible carbon sources (such as plant polysaccharides), and production of different metabolites (such as vitamins and short-chain fatty acids), among which butyric acid is of paramount importance for colonocyte physiology (3). Despite of the growing number of solid scientific evidences showing that our intestinal microbiota is implicated in maintaining the gut physiology, we are far from reaching a clear understanding about the molecular mechanisms underlying this mutualism and how alterations in the relative amounts of microbiota populations are related to human health (4).

In order to maintain intestinal homeostasis, host bacteria and human immune system are constantly engaged in sensing and reacting to one another by means of specific molecules such as extracellular proteins (5). The human gastrointestinal tract (GIT), and more concisely the gut-associated lymphoid tissue (GALT), is perhaps the main point of contact between gut microorganisms and the human host. Peyer’s patches and lymphoid/plasma cells are important GALT structures in the lower parts of the GIT, and whereas the first have precise localizations, the second are diffusely distributed throughout the gut mucosa (6). Interaction between microbiota and host, also referred to as molecular cross-talking, is elicited by a series of relatively well-conserved molecules known as microbial-associated molecular patterns (MAMPs). MAMPS interact with specific receptors on the surface of host cells named pattern-recognition receptors (PRRs), driving the normal interaction occurring in a homeostatic situation and also some of the beneficial effects attributed to probiotics (7). The interaction of MAMPs with PRRs also affects the maturation of antigen-presentation cells, which will subsequently define the type of T-cell immune response (mainly Th1, Th2, Th17 or Treg) (8).

Several non-infectious human diseases, including autoimmune, inflammatory diseases and certain types of cancer, are related to both an immunological imbalance and a dysbiosis of the gut microbiota (9–12). Possibly, the paradigm of the link between gut microbiota and immunity are the Inflammatory Bowel Diseases (IBD), in which an aberrant immune response to the gut microbiota is related to development, persistence and relapse of the disease (13, 14). Indeed, faecal microbiota transplantation leads to complete remission in about 40% of the cases, even though there is a relative high heterogeneity among patients (15). In this regard, some species have been found to be underrepresented in the faecal microbiota of patients suffering from IBD, such as the butyrate producing bacteria *Faecalibacterium praustnizii*, which has been postulated as a biomarker or even as a potential therapeutic agent (16, 17). However, it should be taken into account that several studies point to the involvement of genetic predisposition due to IBD-promoting gene mutations (18, 19).

This work introduces the Mechanism of Action of the Human Microbiome (MAHMI) database, a novel and comprehensive resource containing peptides extracted from the human microbiome complemented with potential immunomodulatory or antiproliferative bioactivity. MAHMI has two data sections: the reference data section that contains peptides whose bioactivity has been experimentally tested and the *in silico* data section, which keeps peptides encrypted in larger bacterial proteins that exhibit sequence similarity with the peptides contained in the reference data section, and which might be potentially bioactive. Our bioactivity prediction method encompasses *in silico* protein digestion using intestinal proteases from protein microbiome complements and global sequence similarity search against the reference peptides.

The overall aim of MAHMI is to facilitate the work of those scientific teams working in the field of the molecular interaction between the human host and the intestinal microbiota. Most notably, MAHMI Web-based interface enables the comparison of user-submitted amino acidic sequences against database contents and thus, it can be ideally used to identify peptides that may be used to manipulate altered immune responses. MAHMI has a clear application in the framework of IBD, but also in other immune-associated diseases where the gut microbiota plays a part, including Irritable Bowel Syndrome (20, 21), Celiac Disease (22, 23) and Type-1 Diabetes (24, 25).

## Database construction and update

### Database overview

The system integrates a Web-based front-end, a Java application as processing core (version 1.8.0_66) and a MySQL data management system (version 14.14). The front-end is a PHP application (version 5.5.9) with a Bootstrap interface. The MAHMI site is best viewed by Google Chrome 46.0, Firefox 45.0.1, Opera 36.0 and Safari browsers.

### Data sources: reference and *in silico* sections

**Figure 1.**
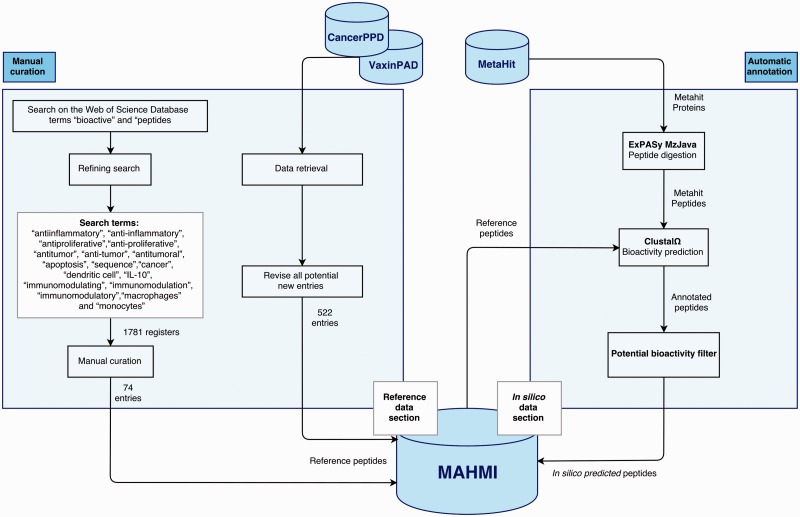
Data sections and curation workflow. The curation workflow is divided into two processes: manual curation and automatic annotation.

The MAHMI database consists of two data sections (Figure 1). The *reference* section (left side) is manually annotated and reviewed, containing information extracted from the scientific literature and other public, manually curated databases about experimentally validated immunomodulatory peptides. The *in silico* data section (right side) is automatically generated and annotated based on the computational analysis of the exoproteomes of the human microbiome and the identification of peptides with immunomodulatory potential.

The main sources of the reference section are scientific literature, the VaxinPAD server of peptide-based vaccine adjuvants (26) and the CancerPPD database on antiproliferative peptides and proteins (27). Although retrieval and annotation of some of these data is automated at some extent, this curation workflow is primarily manual. Most notably, scientific literature is retrieved from the Web of Science Database (23/05/2015) and manually curated by experts. Search starts by using the terms ‘bioactive’ and ‘peptides’, and is further refined using terms such as ‘antiinflammatory’, ‘anti-inflammatory’, ‘antiproliferative’, ‘anti-proliferative’, ‘antitumor’, ‘anti-tumor’, ‘antitumoral’, ‘apoptosis’, ‘sequence’, ‘cancer’, ‘dendritic cell’, ‘IL-10’, ‘immunomodulating’, ‘immunomodulation’, ‘immunomodulatory’, ‘macrophages’ and ‘monocytes’. The first literature retrieval rendered a total of 1781 records. Works reporting the amino acid sequence of an antiproliferative or immunomodulatory peptide were kept (74 records). The contents of VaxinPAD and CancerPPD databases are automatically retrieved, but our curators revise all new entries before insertion into MAHMI database. Moreover, all peptide entries in MAMHI maintain cross-links to the original sources (i.e. database identifiers and PubMed identifiers, in the case of scientific literature).

Complementarily, the MetaHIT project (http://www.metahit.eu/) is the primary data source of the *in silico* data section (28). Specifically, protein data downloadable via the GigaDB FTP site (ftp://climb.genomics.cn/pub/10.5524/100001_101000/100064/1.GeneCatalogs/). These proteins are processed and the peptides that show immunomodulatory or anti-inflammatory potential are inserted in MAHMI database. Noteworthy, this data compilation and screening process runs in continuous mode and MAHMI database is routinely updated on a weekly basis.

### *In silico* bioactivity prediction

This module is implemented as a Java 8 application that uses a REST API to communicate with the rest of the system. Our REST API ensures the modular design of the system, such that each component performs a specific/limited scope functionality and hence, individual components may be interchanged as needed without affecting the rest of the system. Notably, communication is based on the interchange of XML and JSON objects. The data workflow is as follows.

#### Protein digestion

This module is responsible for the digestion of the proteins downloaded from MetaHIT. *In silico* digestion is performed using the ExPASy mzJava (http://mzjava.expasy.org) library (29) and considering the major human intestinal endoproteases, i.e. trypsin, chemotrypsin and pepsin (pH < 2). The resulting collections of peptides, denominated peptidomes, are stored in the MySQL data repository.

#### Bioactivity prediction

All peptides generated by enzyme digestion are processed to predict any possible bioactivity. The MAHMI module responsible for this task is built on top of ClustalΩ, the latest and more scalable version of the well-known Clustal multiple sequence alignment tool (30). Peptides are aligned against the peptides of the reference data section (described in the *Data Sources* section).

For the first version of the database and in an attempt to give a first bioactivity index, a peptide was considered as potentially bioactive if its amino acid similarity with experimentally validated peptides was >60% as returned by the ClustalΩ algorithm.

### Query capabilities

The graphical user interface (GUI) of MAHMI database is available at http://mahmi.org. This is a PHP application with a dynamic and user-friendly Bootstrap interface, which expedites the use of HTML5, CSS3 and JQuery technologies in interface development (http://getbootstrap.com/).

#### Quick search

By selecting this option, the user can compare amino acidic sequences against the two data sections of MAHMI. The list of results is produced based on the similarity scores calculated by the NCBI BLAST+ tool BLASTP alignment (31).

#### Advanced search

**Figure 2.**
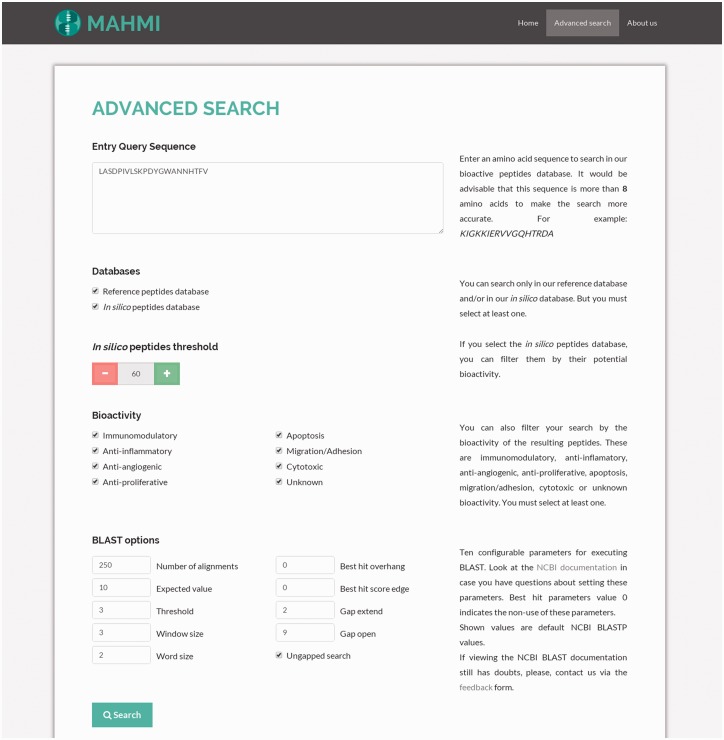
Snapshot of the advanced search. The query sequence is ‘LASDPIVLSKPDYGWANNHTFV’ and both data sections are searched, with the following custom BLAST options: threshold set to 3, window size set to 3, word size set to 2, gap extend set to 2, gap open set to 9 and ungapped search selected.

The configuration of advanced search options (Figure 2) allows the user to select a specific data section and use search facets, such as the bioactivity potential threshold (i.e. predicted peptide should have a similarity equal or greater than the specified threshold) or the bioactivity type (i.e. immunomodulatory, antiproliferative or both). These filters are applied over BLASTP alignment results.

#### Results page

MAHMI Web interface enables the graphical interpretation of all alignments of the user sequence to database records. This interpretation is enabled by two visual and interactive components: (1) a HTML object that presents the distribution of BLAST hits on the query sequence, indicating the extent and region of the alignments; (2) a table that describes the main high-scoring pair attributes of all the hits (32). These two visual components have been built using BlasterJS, an in-house developed and publicly available JavaScript library for displaying BLAST results (http://sing.ei.uvigo.es/blasterjs).

**Figure 3.**
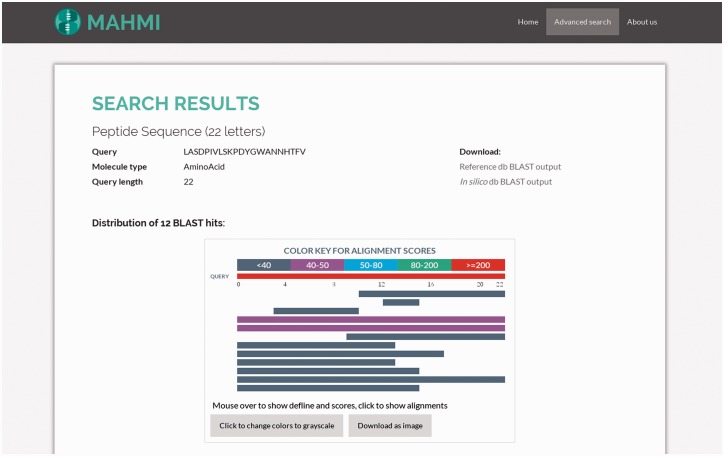
Snapshot of the hit distribution of results for a query search. The query sequence is ‘LASDPIVLSKPDYGWANNHTFV’ and both data sections are searched, with the following custom BLAST options: threshold set to 3, window size set to 3, word size set to 2, gap extend set to 2, gap open set to 9 and ungapped search selected. Returned hits are presented in the same order as they appear in the BLAST file. Also, hits are coloured based on the assigned bit score.

As illustrated in Figure 3, the hits distribution component summarises all search results. The visual appearance and interpretation of this component is similar to that of NCBI BLAST service, which is quite familiar to most domain users (http://blast.ncbi.nlm.nih.gov). Exception being that in our visual component hits coming from the reference data section will be presented first. Hits are coloured by score (full-colour and grey-scale views are available).

**Figure 4.**
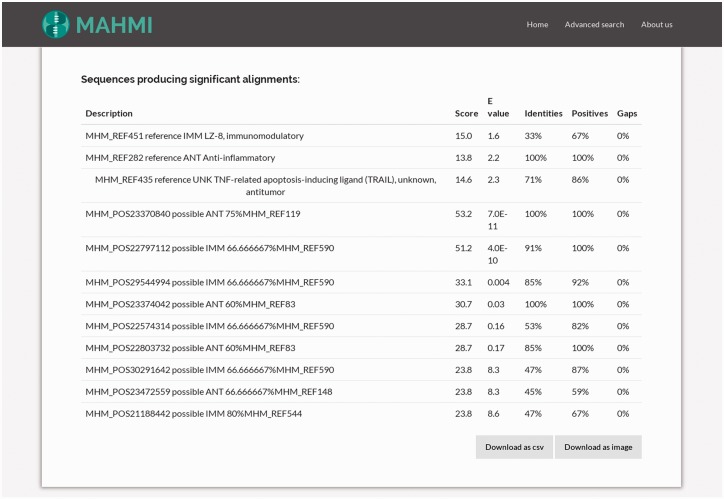
Snapshot of the table detailing the hits of a query search. This table gathers together all BLAST hits and their attributes (bit score, expected value, identities, positives and gaps). Clicking on one hit, it redirects the user to the alignment information of this hit.

By clicking on one hit, the user views the corresponding hit in the alignment table. As illustrated in Figure 4, the alignment hits table gathers together all BLAST hits and their attributes: bit score, expected value (*e*-value), identity, positives and gaps (32). Record ordering is two-fold: first, entries are sorted by data section provenance, i.e. reference entries show first than *in silico* predicted entries; and then, results are sorted by BLAST similarity score.

#### Hit alignment page

**Figure 5.**
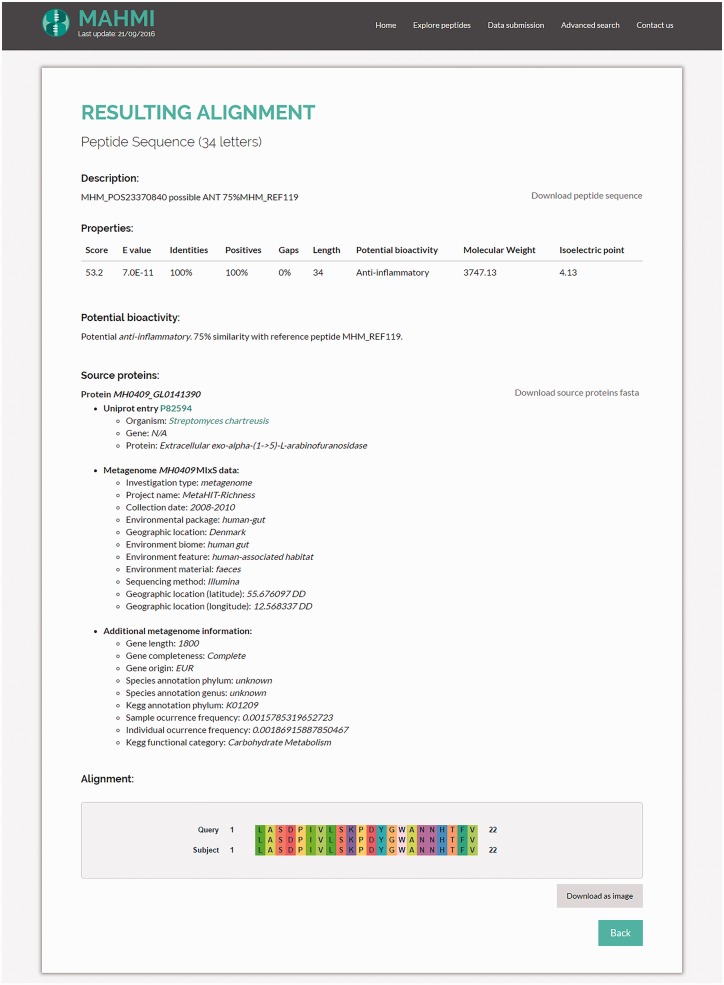
Snapshot of the hit alignment information. Properties table includes the high-scoring pair attributes of the hit, bioactivity (potential bioactivity, in the case of *in silico* predictions), molecular weight and isoelectric point. Source proteins are cross-linked to UniProt and Metahit metagenome entries. The alignment figure compares in more depth (amino acid by amino acid) the alignment of the proposed hit with the query sequence.

By clicking on an alignment description on the alignment hits table (Figure 4), the system shows an in-depth visual representation (amino acid by amino acid) of the alignment (Figure 5). Moreover, the user has access to additional information about the matching peptide, such as sequence length, bioactivity potential (potential or experimentally validated), molecular weight and isoelectric point. In addition to these data, MAHMI records are compliant with the Minimum information about any (x) sequence (MIxS) checklist for metagenomes (34). Whenever available, source proteins are cross-linked to external database records, namely, UniProt (33), CancerPPD (27), VaxinPAD (26) and Metahit metagenome records.

### Results download

All search results, namely the BLASTP alignments, the peptide sequences and all figures can be downloaded. Specifically, the user can obtain the BLASTP output file (default format) as well as the hits distribution component as image (PNG format). The alignment table can be exported as image (PNG format) or plain text file (CSV format). On the alignment information page, the detailed alignment figure can be downloaded as PNG image. The user can also download additional peptide information, namely, sequence and possible sources, whenever available.

### Public REST API

In addition to the graphical user interface, MAHMI provides a public REST API to download all data relating to proteins and peptides in the database. The main methods provided by this API are listed below:

- GET/peptides/reference, which returns the reference peptides;
- GET/peptides that returns the bioactive peptides;
- POST/peptides/search + search XML object (see details in Supplementary material 1), which enables batch searches, similar to those that can be performed on the web;
- GET/peptides/sourceProteins/$peptide_id, which returns information about a given peptide, namely the list of source proteins (cross-referenced to Uniprot) and metagenome information, namely MIxS compliant data;
- GET/proteins/$peptide_id, which returns the proteins that produce a specific peptide.

This API is publicly available at http://mahmi.org/api.

### Database utility

To the best of our knowledge, and with the exception of VaxinPAD and CancerPPD, no effort has been conducted to extensively collect data on peptides encrypted in the protein complement of the human microbiome with immunomodulatory and antiproliferative potential bioactivities. This prompted us to create MAHMI, an online and accessible resource containing massive information on potential bioactive peptides. Sequences contained within MAHMI database may help the basic researcher working in the field of bacteria-human host molecular cross-talk, as this kind of peptides are relevant from a physiological and immune point of view.

Proteins produced by our intestinal microbiota are important molecular targets that can allow the study of the mechanism of action of our gut microbes (35). Notably, surface proteins are key extracellular components in the mediation of certain interactions, such as immunomodulation or mucosal colonization (5). A subset of these proteins, the secreted proteins, may even be able to interact directly with mucosal cells once released by the microorganism to their surrounding environment (36). Extracellular proteins from the gut microbiota are either cleaved by GIT proteases or processed by professional antigen presenting cells such as dendritic cells (37). This interaction mediated by peptides produced by the digestion of our gut microbe proteins ends in physiological changes of the cell (38), including signalling pathway regulation, changes in the secretion of chemokins, cytokines and anti-bactericidal peptides (defensines), mucus secretion, production of pseudopods by dendritic cells (DCs), rearrangement of the tight-junctions in epithelial cells, and modulation of the immune function and the response of the gut-associated lymphoid tissue (GALT) cells (39).

This is a very promising field and, for instance, recent results have revealed that a peptide encrypted in a *Lactobacillus plantarum* extracellular protein, more concisely an aggregation factor, is able to modulate the immune status of both intestinal and monocyte-differentiated DCs, including cytokine secretion and toll-like receptors (TLR) expression (40, 41). By querying the content of MAHMI database, the researcher will easily have insights about the potential bioactivity of the user-submitted amino acid sequence.

## Conclusions

MAHMI database offers a novel and comprehensive repository about peptides with immunomodulatory and antiproliferative potential. MAHMI has the potential to aid in the study of how to balance altered immune responses and, ideally, in the innovative development of new functional ingredients or drugs based on bioactive peptides obtained from the genetic sequences of the human microbiome. These may be directed to the prevention of several gastrointestinal disorders of autoimmune and inflammatory nature as well as the minimisation of the effects of the disease in patients.

## Availability and requirement

The Mechanism of Action of the Human Microbiome (MAHMI) database is freely accessible for research purposes for non-profit and academic organisations at http://mahmi.org/.
